# The Enemy Which Sealed the World: Effects of COVID-19 Diffusion on the Psychological State of the Italian Population

**DOI:** 10.3390/jcm9061802

**Published:** 2020-06-10

**Authors:** Giuseppe Forte, Francesca Favieri, Renata Tambelli, Maria Casagrande

**Affiliations:** 1 Department of Psychology, “Sapienza” University of Rome, 00185 Rome, Italy; francesca.favieri@uniroma1.it; 2 Department of Dynamic and Clinical Psychology, “Sapienza” University of Rome, 00185 Rome, Italy; renata.tambelli@uniroma1.it

**Keywords:** COVID-19, pandemic, anxiety, psychopathological symptomatology, mood, post-traumatic stress disorder (PTSD), emergency

## Abstract

Background: Starting from the first months of 2020, worldwide population has been facing the COVID-19 pandemic. Many nations, including Italy, took extreme actions to reduce the diffusion of the virus, profoundly changing lifestyles. The Italians have been faced with both the fear of contracting the infection and the consequences of enforcing social distancing. This study was aimed to understand the psychological impact of the COVID-19 outbreak and the psychopathological outcomes related to the first phase of this emergency. Methods: The study included 2291 respondents. An online survey collected information on socio-demographic variables, history of direct or indirect contact with COVID-19, and additional information concerning the COVID-19 emergency. Moreover, psychopathological symptoms such as anxiety, mood alterations and post-traumatic symptomatology were assessed. Results: The results revealed that respectively 31.38%, 37.19% and 27.72% of respondents reported levels of general psychopathological symptomatology, anxiety, and PTSD symptoms over the cut-off scores. Furthermore, a significant worsening of mood has emerged. Being a female or under the age of 50 years, having had direct contact with people infected by the COVID-19, and experiencing uncertainty about the risk of contagion represent risk factors for psychological distress. Conclusions: Our findings indicate that the first weeks of the COVID-19 pandemic appear to impact not only on physical health but also on psychological well-being. Although these results need to be considered with caution being based on self-reported data collected at the beginning of this emergency, they should be used as a starting point for further studies aimed to develop interventions to minimize both the brief and long-term psychological consequences of the COVID-19 pandemic.

## 1. Introduction

In December 2019, an outbreak of pneumonia associated with a new coronavirus (i.e., severe acute respiratory syndrome due to coronavirus 2 (SARS-CoV-2)) was reported in Wuhan, China. In the following weeks, the infection attracted worldwide attention for its rapid and exponential diffusion across different countries around the world. On 12 February 2020, WHO named it Coronavirus Disease 2019 (COVID-19) [1].

At the beginning of April 2020, COVID-19 has infected more than one and a half million people, causing over 80,000 deaths in 204 countries [1]. This viral infection spread quickly, becoming unstoppable, and forcing the WHO to declare it a pandemic [1]. Although the containment measures implemented in China have been successful in the reduction of new cases by more than 90%, this trend was not reported in other countries, including Italy. According to the Italian Institute of Health, Italy, until 8 April 2020, has had 139,442 confirmed cases of infection and 17,699 deaths, becoming one of the countries with the highest rate of death due to the COVID-19 outbreak [2]. On 8 March 2020, the Italian Government adopted extraordinary measures to limit viral transmission, minimizing contacts with people infected by the virus. The Italian population has been subjected to a period of forced social distancing, with restricted movements. It is the first time in Italy that such restrictive measures have been taken to contain the spread of infection. These actions had a high impact on the Italian lifestyles (e.g., working, education, social interactions). However, there are worldwide precedents for these measures. For example, during the 2003 outbreak of severe acute respiratory syndrome (SARS) in China and Canada, or during the 2014 Ebola occurrence in Africa [3], quarantine and social distancing rules were also imposed.

Recent reviews suggest that the psychological impact of quarantine and social distancing is wide-ranging, substantial, and can be long-lasting, including anxiety and mood disorders, psychological distress and post-traumatic stress disorder, sleep disturbance, and other psychopathological conditions [3,4]. Accordingly, as reported by previous studies on the COVID-19 emergency in China [5,6,7,8,9], we aimed to investigate the psychological status of the Italian people in the early stages of the COVID-19 outbreak, trying to define the reaction of the Italians to the government’s measures of enforced social distancing in this extraordinary situation. Specifically, we focused our attention on the level of anxiety, mood, and other psychopathological symptoms as indicators of general distress in the current conditions. We also tried to identify possible differences in the Italian territorial areas (North, Central and South Italy) as a consequence of the heterogeneous diffusion of the contagion that has seen North Italy as the central core of the emergency, with the highest number of infections and deaths due to COVID-19.

Moreover, we tried to evaluate mood changes by comparing participants’ self-perception of mood before and after the spread of the infection.

## 2. Methods

### 2.1. Study Design and Participants

A web-based cross-sectional survey, implemented using the Kobo Toolbox platform and broadcasted through mainstream social-media (such as Facebook, Twitter, Instagram, Telegram), was used to collect data among the Italian speaking population. In our opinion, this procedure represents the best data collection strategy in the present phase of forced social distancing, and it leads to reaching the largest number of people. The survey was carried out from 18 March 2020 to 31 March 2020. A brief presentation informed the participants about the aims of the study, and electronic informed consent was requested from each participant before starting the investigation. The survey took approximately 30 min to complete. When the participants’ responses to the survey lasted less than 5 min or more than 60 min, data were excluded to ensure a standard quality of questionnaires. Participation was entirely voluntary and free of charge. To guarantee anonymity, no personal data, which could allow the identification of participants, was collected. For the current research, being at least 18 years old was the only inclusion criterion employed.

After a short demographic questionnaire, the participants answered questions that assessed knowledge and perceptions related to the spread of COVID-19 and the government measures adopted to contain it. Finally, Italian versions of standardized questionnaires were administered to assess psychological dimensions. This study was conducted in accordance with the Declaration of Helsinki and was approved by the Ethics Committee of the Department of Dynamic and Clinical Psychology of the “Sapienza” University of Rome (protocol number: 0000266). Participants could withdraw from the study at any time without providing any justification, and the data were not saved. Only the questionnaire data that had a complete set of answers were considered. Ninety-eight per cent of the total respondents (2291 out of 2332 people) who started the questionnaires completed the entire survey, and the related data were considered for statistical analyses.

The main demographic characteristics of the sample are shown in Table 1.

### 2.2. Ethical Standards

The authors assert that all procedures contributing to this work comply with the ethical standards of the relevant national and institutional committees on human experimentation and with the Helsinki Declaration of 1975, as revised in 2008.

## 3. Outcomes

### 3.1. Demographic Questionnaire and COVID Related Information

The first session of this questionnaire required information about gender, age, education and occupation, city, and region of origin. The second section aimed to evaluate personal knowledge about COVID-19 diffusion, individual perception of the situation, and lifestyle changes related to government restrictions.

### 3.2. Symptom Checklist-90 (SCL-90)

The SCL-90 [10] (Italian Version: 11) is a 90-items questionnaire aimed to assess psychological distress and symptomatology. The items are rated on a five-point Likert scale, ranging from ‘not at all’ (0) to ‘extremely’ (4). Ten primary symptom dimensions are measured: Somatization, Obsessive-Compulsive, Interpersonal Sensitivity, Depression, Anxiety, Anger-Hostility, Phobic Anxiety, Paranoid Ideation, Psychoticism, and Sleep Disturbance. A Global Severity Index provides measures of overall psychological distress. Higher scores in each dimension indicate greater distress and psychopathological symptomatology. A cut-off score of 0.90 was selected to define higher psychopathological symptomatology, in line with previous studies on the general Italian population [11,12]. The internal consistency in the participants of the present study was α = 0.97.

### 3.3. State-Trait Anxiety Inventory (STAI-Y)

The STAI measures state and trait anxiety [13] (Italian Version: 14). The questionnaire includes 40 items. Twenty items refer to state anxiety (STAI-S) and evaluate how participants feel about anxiety “right now, at this moment”; 20 items refer to trait anxiety (STAI-T) and assess how people “generally feel” about anxiety. The items are rated on a four-point Likert scale, ranging from 1 (not at all) to 4 (very much so). In both the State and Trait anxiety scales, higher scores indicate greater anxiety levels. A cut-off point of 55 was used to define higher state anxiety, according to Kvaal et al. [14]. Although this study was interested in assessing state anxiety, trait anxiety was also measured to check whether the anxious state could be explained by a high anxious trait of the Italian population. The internal consistency of STAI in the sample of this study was adequate (α = 0.60).

### 3.4. Mood Scales

Fifteen mood aspects (insecurity, helplessness, sadness, fear, anger, frustration, stress, anxiety, depression, boredom, serenity, happiness, preoccupation, tranquility, energy) both positive and negative were assessed to examine the emotional impact of the current situation. In these evaluations, the participant was required to refer to two different periods. The first was December, preceding the outbreak of the contagion (December 2019); the second period referred to the last week. The mood scales required a response on a 10-point Likert scale [15], from 0 (not at all) to 10 (very much). The use of mood scales has mainly been adopted to analyse the self-reported conditions of individual mood [16,17,18]. The items on the Mood Scales presented high internal consistency (α = 0.75).

### 3.5. Impact of Event Scale- Revised(IES-R)

The IES-R is a self-report measure designed to assess PTSD symptomatology according to the Diagnostic and Statistical Manual of Mental Disorders—Fourth version (DSM-IV) criteria for PTSD. The questionnaire requires the indication of the magnitude of distress on specific dimensions (e.g., recurring dreams, feelings of anger and irritability) related to specific life events (i.e., the current COVID-19 emergency) referring to the last seven days [19] (Italian Version: 20). The three subscales measure Avoidance (the tendency to avoid thoughts or reminders about the incident), Intrusion (difficulty in staying asleep, dissociative experiences similar to flashbacks), and Hyperarousal (irritated feeling, angry, difficulty in sleep onset). The IES-R requires a response on a 5-point Likert-scale, from 0 (not at all) to 4 (extremely). The score on an IES-R subscale is the mean of the scores of the items of that cluster. The IES-R also gives an overall score (IES-R total that is the sum of the scores of the three subscales). The cut-off of 33 was adopted to indicate a high risk of PTSD symptomatology [20,21]. In the present sample, the IES-R presented high internal consistency (α = 0.95).

### 3.6. Statistical Analysis

Descriptive analyses were conducted to describe demographic characteristics, and COVID-19 related aspects in the Italian population, considering the different Italian territorial areas. Student’s t-test was performed to compare our data on anxiety, general psychological symptomatology, and PTSD symptomatology with data from the general Italian population, reported by previous studies. Specifically, our data on anxiety were compared with those reported by Corno et al. [22], SCL-90 outcomes were compared with the data given by Holi et al. [12], and PTSD indices were compared with the results of Ashbaugh et al. [23].

Analyses of Variance (ANOVAs) were performed to explore the potential difference in the impact of COVID-19 in the Italian territorial areas. The differences between North Italy, Central Italy, and South Italy were reported for State and Trait Anxiety, psychopathological symptomatology (Somatization, Obsessive-Compulsive, Interpersonal Sensitivity, Depression, Anxiety, Anger-Hostility, Phobic Anxiety, Paranoid Ideation, Psychoticism, and Sleep Disturbance), and PTSD symptomatology (IES-R). Furthermore, within-subjects ANOVA designs were adopted to compare the respondents’ self-reporting mood before and during the COVID-19 emergency.

Logistic regressions were performed to explore the influence of demographic factors and experiences which were COVID-19 related in determining risk for state anxiety (STAI), psychopathological symptoms (SCL-90), and PTSD symptomatology (IES-R).

All data were analyzed using Statistical Package for Social Sciences (SPSS) version 24.0 and Statistica 10.0 (StatSoft.inc., Tulsa, OK, USA). *p*-values of less than 0.05 were considered statistically significant. To better control the results for the multiple comparison analyses, the Bonferroni correction was adopted; in these cases, an adjusted *p*-value of less than 0.01 was considered statistically significant.

## 4. Results

The characteristics of the respondents are shown in Table 1.

Two thousand two hundred ninety-one individuals completed the questionnaires, 580 (25.3%) were males, and 1708 (74.6%) were females; the mean age of the participants was 30.0 years (SD: 11.5 years; age range: 18–89). The most represented age range was 18–29 years (68.6%). Most of the participants (1136; 49.6%) received a high school education and were students (1073; 46.8%) or employees (688; 30.0%). The respondents’ current locations were sorted considering territorial area: North (23.6%), Central (25.1%), and South (51.3%) of Italy. Most of the participants live in urban areas (937; 40.9%) with a number of inhabitants between 10,000 and 100,000.

Among all respondents, only 9 (0.4%) were infected by the COVID-19, and 40 (1.7%) were sure that they had had close contacts with individuals suspected of COVID-19 infection (see Table 1). Of the overall sample, 112 respondents (4.9%) and 177 (7.7%) respectively knew people dead and patients in intensive care units (ICU) because of COVID-19 infection.

Comparisons of state and trait anxiety, psychopathological symptomatology, and post-traumatic symptomatology during the COVID-19 epidemic were made with data from the general population.

The comparisons of psychological outcomes during the COVID-19 epidemic in the Italian population with data from the general population are presented in Table 2.

Considering SCL-90 indices, depression (t = 6.14; *p* < 0.0001), anxiety (t = 7.83; *p* < 0.0001), anger-hostility (t = 1.89; *p* < 0.05), phobic anxiety (t = 9.71; *p* < 0.0001), psychoticism (t = 4.25; *p* < 0.0001), and global severity index (t = 4.18; *p* < 0.0001) significantly differ from Holy’s data [12], indicating greater psychopathological symptomatology in our sample.

Considering STAI indices, state anxiety appears to be higher in our sample compared to data reported by Corno et al. [22] in an Italian sample that considered the levels of anxiety separately in both males and females (males: t = 4.49; *p* < 0.0001; females: t = 9.64; *p* < 0.0001), while no significant differences were present considering trait anxiety.

Finally, PTSD related symptomatology assessed by the IES-R resulted higher in our sample compared to the data reported by Ashbaugh et al. [23] (t = 2.41; *p* < 0.05) (see Table 2).

### 4.1. The Difference in Psychological Outcomes between North, Central, and South Italy

Table 3 reports the differences in psychological outcomes, considering the three territorial areas of Italy.

Considering psychopathological symptomatology assessed by the SCL-90, significant differences were reported only in the sleep disturbance subscale (F_2,2288_ = 4.55; *p* < 0.01; pη^2^ = 0.004). People from North Italy reported higher sleep disturbances compared to people from South Italy (*p* < 0.003). However, no other significant differences were observed (see Table 3).

ANOVAs on STAI subscales did not highlight significant differences between individuals from North, Central, and South Italy.

Finally, considering PTSD, no significant differences were reported in IES-R subscales (see Table 3).

### 4.2. The Impact of the COVID-19 Emergency on Self-Reported Mood

**Figure 1:**
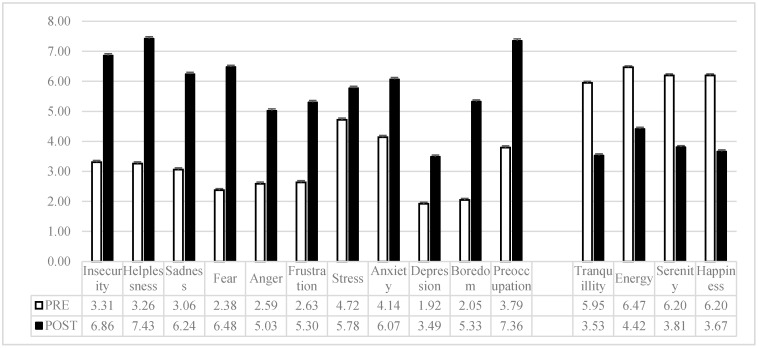
Means and standard errors of self-reported mood before and during the COVID-19 emergency.

The results on the difference in subjective mood before and during the COVID-19 epidemic are shown in Table 4 and Figure 1. The analyses confirmed for all dimensions a perceived worsening of mood by the respondents.

### 4.3. Prevalence and Risk Factors of Psychological Distress during the COVID-19 Pandemic

**Figure 2:**
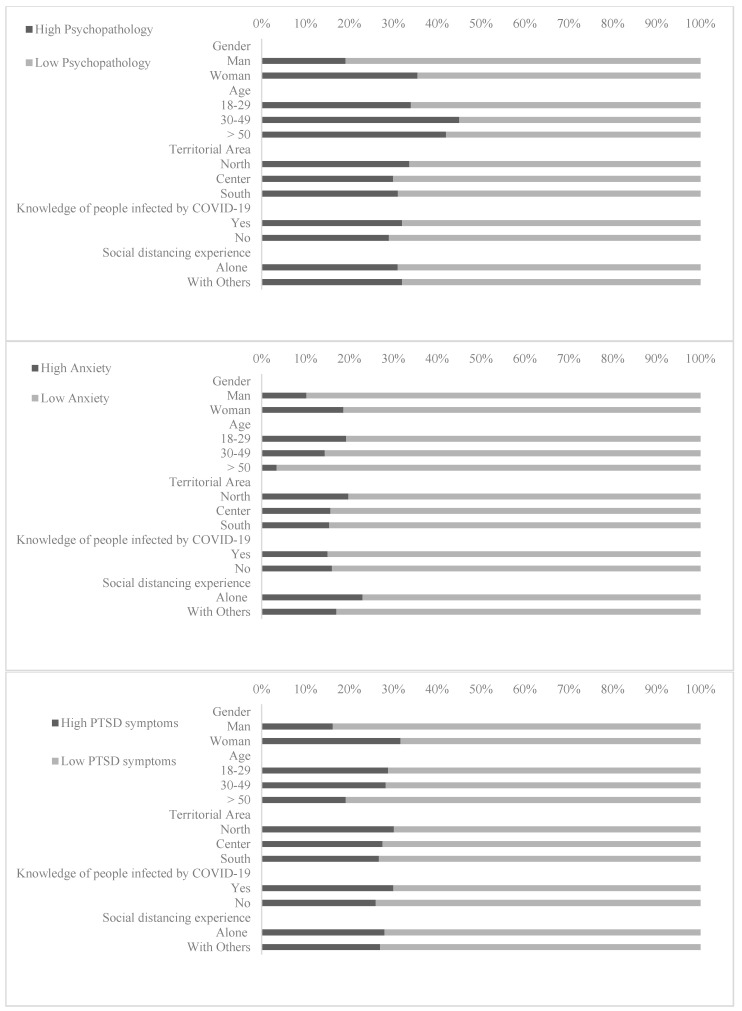
The prevalence of psychopathological symptomatology, state of anxiety, and PTSD, stratified by gender, age, territorial areas, knowledge of people affected by COVID-19, and loneliness in social distancing experience.

Figure 2 shows the prevalence of psychopathological symptomatology, state of anxiety, and PTSD, stratified by gender, age, territorial areas, knowledge of people affected by COVID-19, and loneliness in social distancing experience.

The prevalence of psychopathological symptomatology was 31.38% for the SCL-90, 37.19% for state anxiety assessed by the STAI, and 27.72% for PTSD symptomatology assessed with the IES-R.

Logistic regressions showed that the risk of developing psychopathological symptomatology was higher in females (OR = 2.32; 95% CI = 1.85–2.92), in people younger than 50 years (OR > 1.68), in individuals that felt uncertainty about the possibility of contracting the COVID-19 infection (OR = 1.29; 95% CI = 1.06–1.58) or about the possibility to have direct contact with people infected by COVID-19 (OR = 1.33; 95% CI = 1.10–1.59) and in people who knew infected people (OR = 1.25; 95% CI = 1.02–1.53) or people who died due toi COVID-19 (OR = 1.62; 95% CI = 1.10–2.39). The risk of developing anxiety was higher in females (OR = 3.10; 95% CI = 2.47–3.89), in individuals younger than 50 years (OR > 1.47), in undergraduates (OR =1.68; 95% CI = 1.05–2.68), in postgraduates in health care professions (OR = 3.00; 95% CI = 1.22–7.39), and in people uncertain regarding the possibility of being infected by COVID-19 (OR = 1.29; 95% CI = 1.06–1.56) or in persons uncertain about the possibility of having had direct contact with people infected by COVID-19 (OR = 1.30; 95% CI = 1.09–1.55). Higher risk of PTSD symptomatology was associated with females (OR = 2.39; 95% CI = 1.88–3.05); being aged between 18 and 49 years (OR > 1.66); having uncertainty regarding the possibility of contracting the infection (OR = 1.22; 95% CI = 0.99–1.50); the possibility of having had direct contact with people infected by COVID-19 (OR = 1.32; 95% CI = 1.09–1.59); having known infected people (OR = 1.34; 95% CI = 1.09–1.66) o4 people hospitalized in ICU (OR = 1.45; 95% CI = 1.00–2.00) or who had died due to COVID-19 (OR = 1.88; 95% CI = 1.28–2.77) (See Table 5).

## 5. Discussion

Sudden outbreak events always pose huge challenges to the countries where they occur, impacting not only on physical health but also on social and mental well-being. From this perspective, the COVID-19 pandemic will have long-term consequences, influencing international and national public health policies.

This study is part of a series of works aimed at investigating the characteristics and the psychological effects of the COVID-19 pandemic and the restrictive measures adopted by the Italian Government during the early and more severe stages of the COVID-19 outbreak [24,25]. Since the outbreak of the COVID-19 epidemic, the Italian Government imposed a lockdown in North Italy, expanding it nationwide following the exponential diffusion of the pandemic from the Northern territorial areas to both the Central and South areas. These severe limitations included the request for both people infected by the virus and healthy citizens to isolate themselves at home, prohibiting all other than indispensable activities, and making it mandatory to wear surgical masks to enter public places. Our data were collected near the infection peak (between the end of March and the beginning of April 2020) [2], and they provide an accurate snapshot of Italians’ perception of this emergency.

This study delivers further information to add to the findings reported on the Chinese population that was the first to be severely affected by COVID-19 [5,6,7,8,26], indicating that the effects of this pandemic on the psychopathological conditions are similar in the Italian and Chinese populations. In both countries younger age, student status, female gender and direct contact with COVID-19 infection are associated with a greater psychological impact of the emergency, involving many psychopathological dimensions (e.g., anxiety, distress, sleep disturbance) [5,6,7,8,9,26].

One of the aims of the study was to analyse the psychological impact of the COVID-19 outbreak in the different Italian territorial areas. North Italy was the first area in Italy infected by the COVID-19 and in which social distancing was imposed. It continues to have the highest prevalence of contagion and deaths, with a heavy burden on the public health system. Accordingly, we expected an impact of these conditions on the psychological well-being and mental health of its inhabitants. However, although respondents from North Italy reported more sleep disturbances and a relatively higher state of anxiety compared to those from Central and South Italy, no other differences were observed in psychopathological symptoms and PTSD risk [23]. These results would seem to underline that psychological status is not only influenced by the direct effects of a justifiable fear of contagion but also by the indirect consequences of the COVID-19 outbreak such as the restrictive measures, that equally influenced people of all the Italian regions, generating a similar psychological pattern. This assumption would be confirmed by the comparison of our results with data from the general Italian population. The differences in the selection of the sample do not allow a generalizability of these results. Most of the psychological symptoms assessed by the SCL-90 subscales are significantly higher in our sample compared to data from the general population. Only somatization and paranoid ideation resulted in being not significantly different from data on the general population. These last findings do not agree with recent data on the Chinese population [27], and they could appear incongruous because medical emergencies might induce higher somatization and intrusive and threatening thoughts. However, these results concord with those found during the SARS epidemic [28].

The high prevalence of anxiety evidenced in our sample highlights that the COVID-19 pandemic has increased alert levels and generated a high level of state anxiety in the population, confirming results of previous studies on SARS, Influenza A virus subtype H1N1 [29,30,31], and COVID-19 [6,7,8].

In our sample, 27.72% of the respondents presented PTSD symptomatology, and risk of PTSD higher than that reported in the general population, at least as regards the symptoms evaluated with the IES-R questionnaire [23]. This result should be interpreted with caution because it referred to the first weeks of the emergency when people could perceive the rapid spread of the virus and the extraordinary measures adopted by the Government as sudden stressors, and it is known that sudden stressors affect the daily lives of individuals drastically. On the other hand, this first Italian perception of the current situation would seem to give a photograph of the real impact of the COVID-19 outbreak on mental health.

Another interesting result concerns the impact of the pandemic on mood. Respondents perceived a significant change in their mood, with a sensitive decrease of positive mood (e.g., happiness, serenity) and a high increase of negative mood (e.g., sadness, preoccupation, boredom) after the COVID-19 spread and the consequent social distancing measures. From a clinical point of view, this result could suggest a possible risk of mood disorders, such as depression, as long-term consequences of a pandemic [32]. However, it must be underlined that these data are not obtained prospectively, and the causal relationship cannot be confirmed. Self-reported moods are subject to memory distortions and bias, and they should be taken with caution.

Overall, the results highlighted high levels of anxiety, psychopathological symptoms and PTSD symptoms in Italian respondents during the first critical phase of the spread of the COVID-19 pandemic and of the Government measures taken to contain it.

However, the results of the present study also suggested which people are most vulnerable to the psychological consequences of the COVID-19 outbreak. This unexpected situation seems to have had a higher impact on females and people under 50 years. Moreover, to have had direct contact with people infected by the virus, and to know people more or less severely infected by the COVID-19 (i.e., people hospitalized in an intensive care unit or people dying as consequences of COVID-19 infection) emerged as other relevant risk factors for psychological well-being. All these characteristics would make people more vulnerable to developing anxiety, psychopathological symptoms, and PTSD-related symptoms, confirming results observed in previous studies [8,33]. These risk factors may depend on different aspects of the COVID-19 pandemic. The high psychopathological risk related to direct experience with the COVID-19 infection could depend on the fear of contagion, while being younger could be a risk factor due to the sense of constraint caused by social distancing and the other measures taken by the Italian Government [3].

Our study reports that COVID-19 infected 0.4% of the sample. This result is higher than the data on the general Italian population (0.22%), updated on the 30 March 2020 [2], but it indicates the high rate of healthy individuals in the sample. Both this consideration and the data on risk factors would confirm that, even without real exposure to the COVID-19 and an actual infection, fighting against an invisible enemy could affect mental health. Uncertainty, fear about infection and social consequences of a pandemic could be triggers for psychopathological symptoms, and they should be considered in further studies.

Although some psychological characteristics are linked to medical conditions [34,35,36,37], psychological consequences of at-risk people are often overlooked during an epidemic emergency as reported for SARS and H1N1 [29,30,33]. Once again, the importance of not disregarding mental health and intervening during and after the pandemic emergency in the most affected psychological dimensions appear relevant in a long-term perspective.

This study gives a picture of the psychological well-being of the Italian population at the beginning of the COVID-19 emergency. However, some limitations must be considered. Despite the large sample size, it is not possible to overcome the limitation of a cross-sectional study, which does not allow us to determine a causal relationship between the variables. Also, the use of an online survey presents other limitations. Selection bias of participant recruitment is a consequence of this methodological choice. This bias is expressed by some characteristics of our sample, such as the higher number of respondents younger than 30 years, and the high number of females and people from South Italy. Another limit related to the online survey can be associated with convenience sampling that may have induced the collection of responses primarily from people who feel strongly about the considered issue. These limitations reduce the representativeness of our findings and may have influenced the results of the study. Therefore, they must be considered. However, the adoption of an online survey was the best solution in this emergency in which social distancing measures limit data collection.

In conclusion, a global response is desperately needed to prepare health systems to face the new challenge of the COVID-19 outbreak. Despite the underlined limitations, these preliminary findings, in line with the results of previous studies, evidenced that the diffusion of this pandemic can be related to anxiety, changes in mood, high psychopathological symptomatology, and could be associated with the development of PTSD. Moreover, similarly to the results of other studies on the COVID-19 pandemic, these findings should be considered preliminary, but they can be useful to predispose interventions aimed at improving the psychological conditions of the population. Generally, there is still a lack of relevant research on psychological aspects during the COVID-19 epidemic. It would be essential to analyse further psychological dimensions related to the COVID-19 outcomes, such as lifestyle changes, fear, and perception of the emergency, to assess their role in influencing the psychological status of the Italian population.

We hope that these preliminary data can be useful to other researchers in analysing the impact of the infection and social isolation due to COVID-19 diffusion. It is our desire that COVID-19 be defeated but also that the research on this topic grows so that we can start thinking about the mental health of those involved in this severe emergency.

## Figures and Tables

**Table 1:** Demographic characteristics of the sample and their distribution in the Italian territorial areas.

	Overall Sample (*n* = 2.291)	North Italy (*n* = 541)	Central Italy (*n* = 574)	South Italy (*n* = 1.176)
Gender, *n* (%)				
Male	580 (25.3)	107 (18.4)	121 (20.9)	352 (60.7)
Female	1708 (74.6)	434 (25.4)	451 (26.4)	823 (48.2)
Other	3 (0.1)	-	2 (66.7)	1 (33.3)
Age, *n* (%)				
18–29 years old	1571 (68.6)	342 (21.8)	374 (23.8)	855 (54.4)
30–49 years old	485 (21.2)	156 (32.2)	130 (26.8)	199 (41.0)
>50 years old	235 (10.3)	43 (18.3)	70 (29.8)	122 (51.9)
Education, *n* (%)				
Until middle School	99 (4.4)	22 (22.2)	18 (18.2)	59 (59.6)
High School	1136 (49.6)	265 (23.3)	242 (21.3)	629 (55.4)
Undergraduate				
Health care	246 (10.7)	49 (19.9)	80 (32.5)	117 (47.6)
Other	660 (28.8)	174 (26.4)	165 (25.0)	321 (48.6)
Post-graduated				
Health care	63 (2.7)	10 (15.9)	28 (44.4)	25 (39.7)
Other	87 (3.8)	21 (24.1)	41 (47.1)	25 (28.7)
Occupation, *n* (%)				
Student	1073 (46.8)	207 (19.3)	272 (25.3)	594 (55.4)
Employed	688 (30.0)	227 (33.0)	162 (23.5)	299 (43.5)
Unemployed	279 (12.2)	52 (18.6)	61 (21.9)	166 (59.5)
Self-Employed	222 (9.7)	50 (22.5)	64 (28.9)	108 (48.6)
Retired	29 (1.3)	5 (17.2)	15 (51.7)	9 (31.1)
Number of inhabitants in own city, *n* (%)				
<2.000	124 (5.4)	28 (22.6)	17 (13.7)	79 (63.7)
2.000–10.000	453 (19.8)	130 (28.7)	81 (17.9)	242 (53.4)
10.000–100.000	937 (40.9)	199 (21.2)	174 (18.6)	564 (60.2)
>100.000	777 (33.9)	184 (23.7)	302 (38.9)	291 (37.5)
Quarantine Experience, *n* (%)				
Alone	234 (10.2)	74 (31.6)	59 (25.2)	101 (43.2)
Others	2.057 (89.8)	467 (22.7)	515 (25.0)	1.075 (52.3)
Infection by the virus				
Yes	9 (0.4)	2 (22.2)	2 (22.2)	5 (55.6)
No	1707 (74.5)	374 (21.6)	409 (23.6)	951 (54.8)
Do not know	575 (25.1)	192 (33.4)	163 (28.4)	220 (38.3)
Direct contact with people infected by COVID-19				
Yes	40 (1.7)	28 (70.0)	6 (15.0)	6 (15.0)
No	1441 (62.9)	274 (19.0)	337 (23.4)	830 (58.6)
Do not know	810 (35.4)	239 (29.5)	231 (28.5)	340 (42.0)
Knowledge of people infected by COVID-19				
Yes	550 (24.0)	237 (43.1)	126 (22.9)	187 (30.4)
No	1741 (76.0)	304 (17.5)	448 (25.7)	989 (56.8)
Knowledge of people in ICU due to COVID-19				
Yes	177 (7.7)	87 (49.2)	39 (22.0)	51 (28.8)
No	2114 (92.3)	454 (21.5)	535 (25.3)	1.125 (53.2)
Knowledge of people died due to COVID-19				
Yes	112 (4.9)	66 (58.9)	21 (18.8)	25 (22.3)
No	2179 (95.1)	475 (21.8)	553 (25.4)	1151 (58.2)

**Table 2:** Mean and SD of state and trait anxiety (STAI), psychopathological symptomatology (SCL-90) and post-traumatic symptomatology (IES) outcomes of the responders and comparison with data from the general population.

	Respondents’ Data	General Population’s Data	t Student	*p*
Anxiety (STAI)				
State of Anxiety	Males: 44.28 (11.98) Females: 52.62 (12.06)	Males: 39.03 (10.00) Females: 44.32 (12.75)	t Males: 4.49 t Females: 9.64	*p* Males: <0.0001 *p* Females: <0.0001
Trait of Anxiety	Males: 40.12 (10.80) Females: 44.41 (11.15)	Males: 39.82 (7.62) Females: 45.30 (9.42)	t Males: <1 t Females: 1.44	*p* Males: 0.77 *p* Females: 0.25
Psychopathological Symptomatology (SCL-90)				
Somatization	0.71 (0.71)	0.67 (0.55)	<1	0.32
Obsessive-Compulsive	0.91 (0.78)	0.82 (0.57)	2.04	<0.05
Interpersonal Sensitivity	0.58 (0.64)	0.74 (0.55)	4.36	<0.0001
Depression	1.01 (0.81)	0.73 (0.55)	6.14	<0.0001
Anxiety	0.86 (0.75)	0.53 (0.49)	7.83	<0.0001
Anger-Hostility	0.65 (0.65)	0.58 (0.53)	1.89	<0.05
Phobic Anxiety	0.58 (0.70)	0.24 (0.39)	8.71	<0.0001
Paranoid Ideation	0.57 (0.62)	0.53 (0.58)	1.11	0.26
Psychoticism	0.44 (0.53)	0.31 (0.48)	4.25	<0.0001
Sleep Disturbance	0.37 (0.36)	-	-	-
Global Index Severity	0.74 (0.59)	0.60 (0.44)	4.18	<0.0001
Post-Traumatic Stress Disorder Screening (IES)				
PTSD Total	22.39 (18.08)	20.6 (19.4)	2.42	<0.05

**Table 3:** Mean and SD of state and trait anxiety (STAI), psychopathological symptomatology (SCL-90) and post-traumatic stress symptomatology (IES-R) outcomes in the different Italian territorial areas, and ANOVA’s results.

	Overall Sample	North Italy	Central Italy	South Italy	F	*p*
Anxiety (STAI)						
State of Anxiety	50.51 (12.53)	51.58 (12.72)	50.10 (11.77)	50.21 (12.47)	2.62	0.07
Trait of Anxiety	43.32 (11.21)	43.76 (11.4)	43.40 (10.53)	43.08 (11.15)	<1	0.50
Psychopathological Symptomatology (SCL-90)						
Somatization	0.71 (0.71)	0.73 (0.74)	0.72 (0.69)	0.70 (0.71)	<1	0.68
Obsessive-Compulsive	0.91 (0.78)	0.90 (0.80)	0.88 (0.73)	0.92 (0.79)	<1	0.58
Interpersonal Sensitivity	0.58 (0.64)	0.60 (0.64)	0.55 (0.62)	0.58 (0.66)	<1	0.37
Depression	1.01 (0.81)	1.08 (0.83)	1.01 (0.78)	0.98 (0.82)	2.52	0.08
Anxiety	0.86 (0.75)	0.91 (0.80)	0.84 (0.72)	0.84 (0.75)	1.90	0.15
Anger-Hostility	0.65 (0.65)	0.59 (0.59)	0.65 (0.69)	0.66 (0.64)	2.40	0.10
Phobic Anxiety	0.58 (0.70)	0.59 (0.69)	0.58 (0.71)	0.59 (0.71)	<1	0.90
Paranoid Ideation	0.57 (0.62)	0.54 (0.62)	0.55 (0.62)	0.60 (0.68)	2.10	0.12
Psychoticism	0.44 (0.53)	0.43 (0.50)	0.43 (0.51)	0.43 (0.55)	<1	0.74
Sleep Disturbance	0.37 (0.36)	0.41 (0.38)	0.38 (0.36)	0.35 (0.35)	4.55	<0.01
Global Severity Index	0.74 (0.59)	0.76 (0.59)	0.73 (0.56)	0.74 (0.61)	<1	0.66
Post-Traumatic Stress Disorder Screening (IES-R)						
Intrusion	1.01 (0.91)	1.0 (0.92)	1.03 (0.91)	0.98 (0.90)	1.04	0.35
Avoidance	1.05 (0.83)	1.07 (0.81)	1.05 (0.80)	1.05 (0.85)	<1	0.91
Hyperarousal	0.97 (0.93)	0.99 (0.91)	1.00 (0.91)	0.9 (0.94)	<1	0.57
Total Subscales	3.04 (2.48)	3.11 (2.45)	3.08 (2.43)	2.99 (2.51)	<1	0.61
PTSD Total	22.39 (18.08)	22.91 (17.88)	22.62 (17.72)	22.04 (18.37)	<1	0.61

**Table 4:** Mean and SD of self-reported mood before and during COVID-19 emergency, and ANOVA results.

	Mood before the COVID-19 Emergency	Mood during the COVID-19 Emergency	F_(1,2290)_	*p*	pη^2^
Insecurity	3.31 (2.81)	6.86 (2.62)	2584.89	<0.0001	0.53
Helplessness	3.26 (3.18)	7.43 (2.68)	3018.68	<0.0001	0.57
Sadness	3.06 (2.76)	6.24 (2.72)	2128.68	<0.0001	0.48
Fear	2.38 (2.64)	6.48 (2.74)	3869.14	<0.0001	0.63
Anger	2.59 (2.80)	5.03 (3.29)	1071.69	<0.0001	0.32
Frustration	2.63 (2.81)	5.30 (3.24)	1380.20	<0.0001	0.38
Stress	4.72 (2.91)	5.78 (3.06)	191.53	<0.0001	0.08
Anxiety	4.14 (3.03)	6.07 (3.04)	856.91	<0.0001	0.27
Depression	1.92 (2.55)	3.49 (3.18)	731.68	<0.0001	0.24
Boredom	2.05 (2.51)	5.33 (3.29)	2052.99	<0.0001	0.47
Preoccupation	3.79 (2.72)	7.36 (2.37)	2994.75	<0.0001	0.57
Tranquility	5.95 (2.43)	3.53 (2.42)	1506.60	<0.0001	0.40
Energy	6.47 (2.39)	4.42 (2.59)	1152.18	<0.0001	0.33
Serenity	6.20 (2.40)	3.81 (2.29)	1639.44	<0.0001	0.42
Happiness	6.20 (2.49)	3.67 (2.33)	1992.88	<0.0001	0.47

**Table 5:** Results of logistic regression analyses.

	High Psychopathology			High Anxiety Symptoms			High PTSD		
Prevalence in the overall sample, *n* (%)		719 (31.38)			852 (37.19)			635 (27.72)	
	B	OR (95% CI)	*p*	B	OR (95% CI)	*p*	B	OR (95% CI)	*p*
Gender, *n* (%)									
Male		Reference			Reference			Reference	
Female	0.84	2.32 (1.85–2.92)	<0.0001	1.13	3.10 (2.47–3.89)	<0.0001	0.87	2.39 (1.88–3.05)	<0.0001
Age, *n* (%)									
18–29 years old	0.74	2.10 (1.50–2.95)	<0.0001	0.38	1.47 (1.09–1.98)	<0.01	0.54	1.71 (1.21–2.41)	<0.01
30–49 years old	0.52	1.68 (1.16–2.46)	<0.01	0.52	1.68 (1.20–2.35)	<0.01	0.51	1.66 (1.14–2.43)	<0.01
>50 years old		Reference			Reference			Reference	
Education, *n* (%)									
Until middle School		Reference			Reference			Reference	
High School	0.25	1.28 (0.81–2.02)	0.29	0.52	1.67 (1.07–2.67)	<0.05	0.14	1.15 (0.71–1.85)	0.57
Undergraduate									
Other	0.16	1.18 (0.74–1.88)	0.50	0.52	1.68 (1.05–2.68)	<0.05	0.36	1.43 (0.88–2.33)	0.15
Health Care	−0.07	0.93 (0.55–1.57)	0.78	0.21	1.24 (0.74–2.08)	0.42	−0.13	0.88 (0.51–1.52)	0.65
Post-graduated									
Other	0.13	1.14 (0.61–2.14)	0.68	0.54	1.71 (0.92–3.17)	0.10	0.45	1.56 (0.82–2.96)	0.17
Health Care	−10.00	0.37 (0.16–0.87)	<0.05	0.07	1.07 (0.53–2.16)	0.86	0.06	1.06 (0.51–2.21)	0.87
Occupation, *n* (%)									
Student		Reference			Reference			Reference	
Employed	−0.41	0.67 (0.54–0.82)	<0.0001	−0.13	0.88 (0.72–1.07)	0.20	−0.17	0.85 (0.68–1.05)	0.13
Unemployed	−0.08	0.92 (0.70- 1.21)	0.55	0.20	1.22 (0.93–1.59)	0.15	0.03	1.03 (0.77–1.38)	0.83
Self-Employed	−0.38	0.68 (0.49–0.94)	<0.05	−0.16	0.85 (0.63–1.15)	0.30	−0.20	0.82 (0.59–1.15)	0.25
Retired	−0.96	0.38 (0.15–1.01)	<0.05	−0.15	0.86 (0.40–1.87)	0.71	−0.25	0.78 (0.33–1.84)	0.56
Territorial Area									
North Italy	0.12	1.13 (0.91–1.40)	0.28	0.14	1.15 (0.94–1.42)	0.19	0.17	1.18 (0.95–1.48)	0.14
Central Italy	−0.05	0.95 (0.77–1.18)	0.65	0.03	1.03 (0.84–1.27)	0.77	0.04	1.04 (0.83–1.31)	0.72
South Italy		Reference			Reference			Reference	
Number of inhabitants, *n* (%)									
<2.000		Reference			Reference			Reference	
2.000–10.000	0.34	1.40 (0.91–2.17)	0.13	0.27	1.31 (0.87−1.96)	0.20	−0.07	0.93 (0.60–1.46)	0.76
10.000–100.000	0.13	1.14 (0.75–1.73)	0.54	−0.06	0.94 (0.64–1.39)	0.76	0.04	1.04 (0.70–1.59)	0.84
>100.000	0.09	1.09 (0.72–1.66)	0.69	−0.18	0.83 (0.56–1.23)	0.36	0.03	1.03 (0.68–1.58)	0.88
Quarantine Experience, *n* (%)									
Alone	0.03	0.97 (0.72–1.30)	0.83	−0.27	0.76 (0.57–1.02)	0.06	0.003	1.00 (0.74–1.36)	0.98
Others		Reference			Reference			Reference	
Infection by the virus									
Yes	−0.41	0.67 (0.14–3.22)	0.61	0.82	2.26 (0.60–8.45)	0.23	−1.07	0.34 (0.04–2.74)	0.31
Do not Know	0.26	1.29 (1.06–1.58)	<0.01	0.25	1.29 (1.06–1.56)	<0.01	0.20	1.22 (0.99–1.50)	0.06
No		Reference			Reference			Reference	
Direct contact with people infected by COVID-19									
Yes	0.16	1.17 (0.60–2.29)	0.65	0.32	1.38 (0.73–2.60)	0.32	0.22	1.24 (0.62–2.47)	0.54
Do not Know	0.28	1.33 (1.10–1.59)	<0.01	0.26	1.30 (1.09–1.55)	<0.01	0.27	1.32 (1.09–1.59)	<0.01
No		Reference			Reference			Reference	
Knowledge of people infected by COVID-19									
Yes	0.22	1.25 (1.02–1.53)	<0.05	0.06	1.06 (0.87–1.29)	0.58	0.30	1.34 (1.09–1.66)	<0.01
No		Reference			Reference			Reference	
Knowledge of people in ICU for COVID-19									
Yes	0.23	1.26 (0.92–1.74)	0.16	0.04	0.95 (0.69–1.31)	0.77	0.37	1.45 (1.00–2.00)	<0.05
No		Reference			Reference			Reference	
Knowledge of people died for COVID-19									
Yes	0.48	1.62 (1.10–2.39)	<0.01	0.21	1.23 (0.84–1.81)	0.28	0.63	1.88 (1.28–2.77)	<0.001
No		Reference			Reference			Reference	
